# Experimental Study on Mechanical Properties of High-Ductility Concrete against Combined Sulfate Attack and Dry–Wet Cycles

**DOI:** 10.3390/ma14144035

**Published:** 2021-07-19

**Authors:** Lingling Li, Junping Shi, Jialiang Kou

**Affiliations:** 1 School of Civil Engineering and Architecture, Xi’an University of Technology, Xi’an 710048, China; llling1211@163.com (L.L.); jialiangkou0918@163.com (J.K.); 2 School of Architecture and Civil Engineering, Shangqiu Normal University, Shangqiu 476000, China

**Keywords:** dry–wet cycle, damage variable, high-ductility concrete, mechanical property, sulfate attack

## Abstract

Concrete will deteriorate and damage under sulfate attack.In order to study the degradation characteristics of HDC under sulfate attack, the mechanical properties of high-ductility concrete (HDC) were investigated using the uniaxial compressive strength test of HDC specimens soaked in different concentrations of sulfate solution and subjected to different times of dry–wet cycles. The variations in the compressive strength, loss rate of compressive strength, and the max compressive strength under the action of sulfate attack and dry–wet cycles were analyzed. The analytical expressions of damage variables were given. SEM was used to observe the microstructure of the sample, and the microdamage mechanism of the HDC was explored. The deterioration of the HDC was found to be the result of the combined action of sulfate attack and dry–wet cycles and was caused by physical attack and chemical attack. PVA prevented the rapid development of deterioration. On the basis of the change of compressive strength, the damage variable was established to quantitatively describe the degree of damage to HDC. The experimental results showed that with the increase in the number of dry–wet cycles, the compressive strength of HDC generally increased first and then decreased. As the concentration of the sulfate solution increased, the loss rate of the compressive strength of HDC generally increased and the max compressive strength gradually decreased. With the increase inthe number of dry–wet cycles, HDC first showed self-compacting characteristics and then gradually became destroyed. Compared with ordinary concrete (OC), HDC is superior to OC in sulfate resistance and dry–wet cycles. This study provided a test basis for the engineering application of HDC in sulfate attack and dry–wet cycles environment.

## 1. Introduction

At present, the most widely used building material in the world is concrete, which is also recognized by the engineering community as the material with the most stable compressive strength and safety performance. However, in the sulfate attack and dry–wet cycle environment, concrete is eroded to varying degrees; the volume expansion of erosion products causes micro-cracks at different levels inside the concrete, and the internal structure is gradually damaged, which eventually leads to a decrease in the bearing capacity of the component, a shortened service life and even severe disasters [1,2,3,4]. Salt lakes, saline soil, and collapsible loess are widely distributed in northwestern China. The soil and water in these areas contain different concentrations of sulfate, which causes dissimilar degrees of erosion to bridges, dams, and houses. Worse still, the fluctuating levels of rivers and groundwater with seasonal changes subject concrete to a dry–wet cycle environment, thereby exacerbating the erosion and damage degree of bridges, dams, and houses and seriously affecting their normal service life. Therefore, it is of practical significance to study the mechanical properties of concrete under the combined action of sulfate attack and dry–wet cycles.

High-ductility concrete (HDC) is a new type of fiber-reinforced concrete material developed on the basis of (engineered cementitious composite) ECC theory [5,6,7]. HDC, the abbreviation of engineering cement-based composite material, is a fiber concrete designed by mixing cementitious materials, fine aggregates, water, fibers, and other additives. It abides by the principles of micromechanics and fracture mechanics and considers fiber bridging. It exhibits evident strain-hardening characteristics under tension and high toughness under compression. It overcomes the brittleness of OC and can effectively improve the ductility of the structure; it is characterized by crack arresting and increased density and has favorable application effects on structure reinforcement and anti-seismic features [8,9,10]. The research on different types of concrete subjected to the effects of sulfate attack and dry–wet cycles mainly focuses on certain aspects such as the mechanical properties, transmission characteristics, and mechanism analysis. For example, Luo et al. (2019) concluded that the concrete exhibited different characteristics of physical and chemical attack in high-concentration sulfate and low-concentration sulfate, respectively, according to the triaxial test of concrete under sulfate attack [11]. After adding a 3% NaCl solution into concrete samples and immersing them in 3%, 5%, and 10% Na_2_SO_4_ solutions for internal and external corrosion tests, Zhao et al. (2020) argued that the deterioration of cast-in-place concrete was accelerated under the internal-external combined sulfate attack, mainly because of chemical attack [12]. The above-mentioned scholars have studied the deterioration characteristics and the causes of corrosion of OC under sulfate attack, and they found that the main reason is deterioration caused by corrosion products. Researchersfound that the compressive strength and durability of fiber-reinforced polymers attacked by sulfate were significantly improved under static and cyclic loads [13]. The method of wrapping fiber cloth on the surface of concrete is simple and can increase the strength of concrete to a certain extent, but once the fiber cloth is damaged, it will not have a protective effect. Zhang et al. (2019) revealed that the fundamental reason for ECC’s self-healing recovery mechanical property was the restoration of its fiber bridging ability [14]. Liu et al. discovered that ECC could maintain its durability and high mechanical properties under the combined action of SO_4_^2−^ and Cl^−^ [2]. Chen et al., by conducting an experimental study of concrete eroded by SO_4_^2−^ and Cl^−^ under dry–wet cycles and bending loads, noted that the load accelerated erosion in the tensile zone, while the anti-erosion ability improved in the compression zone [15]. Du et al. (2019, 2017) found that Cl^−^ could not completely control sulfate attack, but that the effect of sulfate attack could be reduced by immersing concrete in a chloride and sulfate solution [16,17]. The bending characteristics of cement-based materials under the combined action of SO_4_^2−^ and Cl^−^ have beenstudied. Cl^−^ has a certain restrictive effect on the sulfate erosion, and there is a lack of research on the mechanical properties of cement-based materials under compressive loads. The distribution of SO_4_^2−^ was studied under different sulfate attack ages, and an effective transmission model for sulfate attack was proposed [18]. Chen et al. analyzed the compressive strength of cement-based materials under the action of sulfate attack and dry–wet cycles and examined the mechanism through a QUANTA250 scanning electron microscope (SEM), an Energy Dispersive Spectrometer (EDS), and an X-ray diffraction (XRD, DY5621/X-pert3) technique; they found that sodium sulfate crystals and gypsum were produced in the hydration products [19]. Under the conditions of dry–wet cycles, suggestions for improving the resistance of recycled concrete to sulfate attack were proposed [20]. Gu et al. tested the 2×2×12 cm^3^ prism with a water–cement ratio of 0.55 in three environments (sulfate attack, delayed ettringite formation, and the coupling effect of both), analyzed the degraded state of specimens by mercury intrusion porosimetry (MIP), and found that sulfate attack and delayed ettringite formation did the most serious damage [21]. Based on X-ray, CT, and grayscale theory, Yuan et al. held that concrete erosion and damage were exacerbated under dry–wet cycles and sulfate attack; moreover, an increase in the concentration of the salt solution increased the severity of the damage [22]. It was pointed out that the development of cracks in the defect area of shotcrete was accelerated under dry–wet cycles and sulfate attack and that its durability was determined by cracks [23]. Wei et al. investigated the deterioration mechanism of concrete under the action of sulfate attack and dry–wet cycles and pointed out that dry–wet cycles could accelerate concrete damage [24]. Alyami et al. concluded that a 10% sodium sulfate solution could obviously accelerate the deterioration of cement-based materials due to physical salt attack compared to a 5% sodium sulfate solution at a concentration of 30% sulfate solution when the mass loss was maximized [25]. The above researchers comprehensively studied the corrosion products and corrosion principles of mortar or concrete under sulfate attack through advanced methods of EDS, SEM, XRD, MIP, etc. but have not done detailed research on the matrix with added fibers under sulfate attack. Özbay et al. explored the dual effects of freezing–thawing cycles and sulfate attack on cement-based composite materials mixed with slag (55%, 69%, and 81% of the total weight of cement) and concluded that the ductility of ECC was significantly reduced, which had nothing to do with the slag content and the freeze–thaw cycles process [26]. High-performance cement-based composites made of gypsum, polypropylene (PP) fibers, ladle slag, citric acid, and other additives were subjected to freeze–thaw cycle tests in the north of Europe under the attack of a combined sodium sulfate chloride solution; the durability of PP fiber increased due to its crack resistance, and its volume increased due to physical and chemical attack [27]. The above-mentioned researchers studied the durability and mechanism analysis of PP fiber concrete and cement-based materials under freeze–thaw cycles and sulfate erosion, but the mechanical properties and erosion mechanism of PVA fibers concrete under sulfate attack and wet-dry cycles are rarely seen.

HDC is a new type of concrete material that developed in the past 10 years. At present, the experimental research on HDC mainly focuses on compression, tension, shear, and fatigue tests [28,29,30,31]. HDC has been applied to engineering projects, such as house reinforcement, high-speed rail track, airport runway, structure of Salt Lake Area, and coastal structures [32,33], in which it often suffers from sulfate attack. The structure of Salt Lake Area and coastal structures are also subject to dry–wet cycles and sulfate attack, causing cracks and affecting service life. Therefore, studying the durability of HDC under the combined effects of sulfate and dry–wet cycles is of great significance for engineering applications. Though a unified theory has not yet been formed, sulfate attack is considered one of the main reasons for the performance degradation of engineering cement-based composite materials [34,35,36]. Therefore, through 40 sets of HDC samples and 40 sets of OC comparison samples, this study comprises a mechanical property experiment under the combined action of sulfate attack and dry–wet cycles to analyze the uniaxial compressive strength, the max compressive strength, loss rate of compressive strength, and appearance characteristics. Based on physics, chemistry, mechanics, and SEM, this study also explores the deterioration mechanism to provide the test basis for the engineering application of HDC in salt lake, saline soil, and collapsible loess areas.

## 2. Experimental Program

### 2.1. Materials

In this study, HDC and OC were made, and 42.5R ordinary Portland cement (according to Chinese Standard GB 175-2007) was adopted. The sand with the fineness modulus of 2.9 was obtained from the Ba River in Xi’an, Shaanxi Province, China, and its maximum particle size was 1.18 mm. In addition, the fineness of the fly ash (45μm square hole sieve residue) wasnot more than 12%. Tap water was used to make specimens. The water-reducing agent was a polycarboxylates superplasticizer. The PVA fiber type was KURALON K-II fiber. The performance indicatorsof PVA are listed in Table 1.The maximum diameter of the stone (coarse aggregate) was 20 mm.To study the erosion of the sample with different concentrations of SO_4_^2−^, pure anhydrous sodium sulfate was used to prepare representative sulfate solutions of different concentrations, with concentrations of 0%, 5%, 10%,and saturation [11,12] (by mass, the concentration at 20°C was 16.3%), of which 0% is a comparative test immersed in water.

### 2.2. Mix Proportion and Specimen Preparation

The mix proportions of HDC and OC in terms of 1L concrete for the mixtures investigated in this study are given in Table 2. The chemical composition of fly ash and cement is shown in Table 3. The sample size was 100mm × 100mm × 100mm. The HDC and OC samples had 40 groups, respectively, each with 3 blocks. The HDC sample used a HJW-60-type forced mixer to mix cement, fly ash, sand, and PVA fiber. The mixture was poured into a standard mold, vibrated, and shaped on a vibrating table. It was removed from the mold after 48 h and placed in a standard curing room (temperature 20 ± 2 °C, relative humidity 97%) for 90 days. The OC sample was made in accordance with GB/T50082-2009 from China, and its standard curing (temperature 20 ± 2 °C, relative humidity 97%) was carried out for28 days.

### 2.3. Experimental Equipment and Procedure

#### 2.3.1. Experimental Equipment

The WAW-1000 universal testing machine was used for the compressive strength test with a maximum load of 1000kN and a stress rate range of 1–60MPa/s. The drying equipment adopted a 101-type electric blast drying oven, and the micro morphology adopted a phenom XL table scanning electron microscope.

#### 2.3.2. Experimental Procedure

The sulfate solution was prepared at room temperature with 0%, 5%, 10%, and saturated concentrations. To ensure its concentration, the sulfate solution was replaced once every 30 days.

The samples were dried to constant weight before the experiment. First, the HDC and OC were immersed in different concentration sulfate solutions for 48 h. They were naturally air-dried for 1 h. Then, they were placed in an oven and continuously dried at 50 °C for 23 h. Afterward, they were cooled to room temperature, forming a complete cycle. The HDC and OC immersed in different concentrations of sulfate solution were subjected to tests for 0, 30, 60, 90, 105, 120, 135, and 150 times of dry–wet cycles. After the sample reached the set age of erosion, the compressive strength test was conducted at a loading rate of 0.6 MPa/s until the sample was destroyed. Finally, the microscopic morphology and structure of the sample were observed and analyzed by SEM.

## 3. Results and Discussion

The initial state refers to the compressive strength of the sample at cured for 28 days (OC) or 90 days (HDC) in a curing room. The sample was not soaked by a sulfate solution. At this time, the number of dry–wet cycles of the sample is defined as 0.

### 3.1. Uniaxial Compressive Strength of HDC

**Figure 1:**
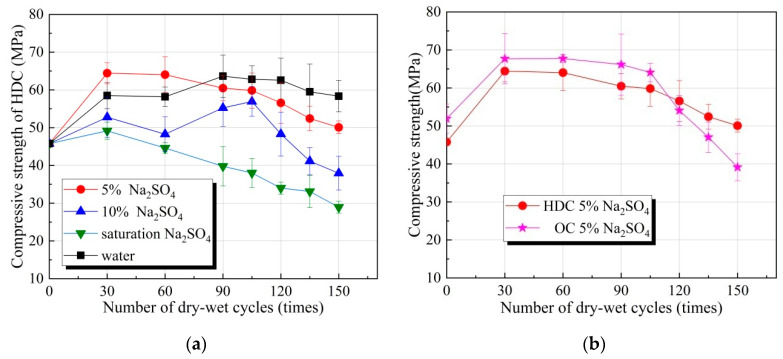

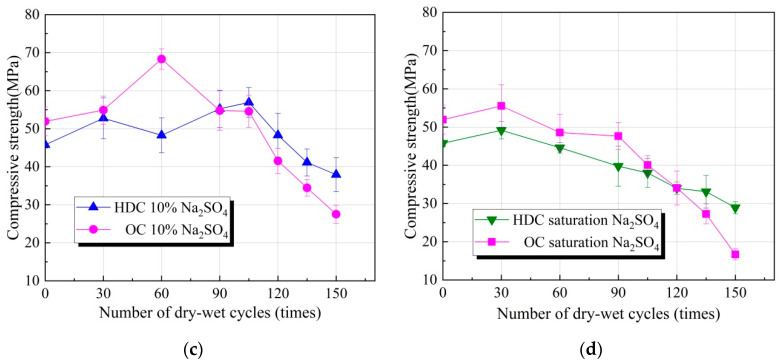
Relationship between compressive strength of HDC or OC and the number of dry–wet cycles in different concentrations of sulfate solution. (**a**) HDC; (**b**) HDC and OC (5% Na_2_SO_4_); (**c**) HDC and OC (10% Na_2_SO_4_); (**d**) HDC and OC (saturation Na_2_SO_4_).

The relationship between the compressive strengths of HDC and OC and the number of dry–wet cycles under different concentrations of sulfate solution are depicted in Figure 1. The result of compressive strengths of HDC and OC under different number of dry–wet cycles under different concentrations of sulfate solution are shown in Table 4 and Table 5. As seen in Figure 1a, with the increase in the number of dry–wet cycles, the compressive strength of HDC first increased and then decreased as a whole. Before 30 dry–wet cycles, the compressive strength of HDC increased due to the increased density of HDC. After 30 dry–wet cycles and before 90 dry–wet cycles, the compressive strength of HDC slowly decreased under the 5% sulfate solution, the compressive strength of HDC first decreased and then increased under the 10% sulfate solution, and the compressive strength of HDC decreased under saturated sulfate solution. After 105 dry–wet cycles, the compressive strength of HDC quickly decreased under the 5% sulfate solution, and the compressive strengths dropped significantly under 10% and saturated sulfate solution, mainly due to a large amount of cracking caused by a large amount of SO_4_^2^^−^ chemical reaction with HDC hydrate. The above discussion shows that the effect of HDC on dry–wet cycles and the 5% sulfate attack was not evident, mainly due to the relatively small amount of SO_4_^2^^−^, and the relatively few chemical reactions occurring, indicating that the damage was severe under the combined action of saturated sulfate attack and dry–wet cycles. As the concentration of the sulfate solution increased, the max compressive strength gradually decreased, indicating that the increase in the sulfate solution concentration could accelerate its internal damage.

As shown inFigure 1b, when the concentration of sodium sulfate solution is 5%, the compressive strength of HDC increases faster than OC before 30 dry–wet cycles. When the number of dry–wet cycles isgreater than 120, the compressive strength of HDC exceeds OC. As shown inFigure 1b–d, the compressive strength of OC decreases faster than that of HDC. Finally, the compressive strength of HDC exceeds the compressive strength of OC. Therefore, the ability of HDC to resist sulfate attack and dry–wet cycles is higher than OC, and the service life will also be greater than that of OC.

In short, as the number of dry–wet cycles increased, the compressive strength of HDC generally increased first and then decreased. At the same time, the increase in the concentration of sulfate solution could accelerate its internal damage. The following comparison shows that HDC was better than OC in resistance to sulfate attack and dry–wet cycles.

### 3.2. Loss Rate of Compressive Strength of HDC

The loss rate of compressive strength of HDC [12,15] (*δ*) was used to characterize the degree of damage of HDC and OC by sulfate attack under the same dry–wet cycles.

**Figure 2:**
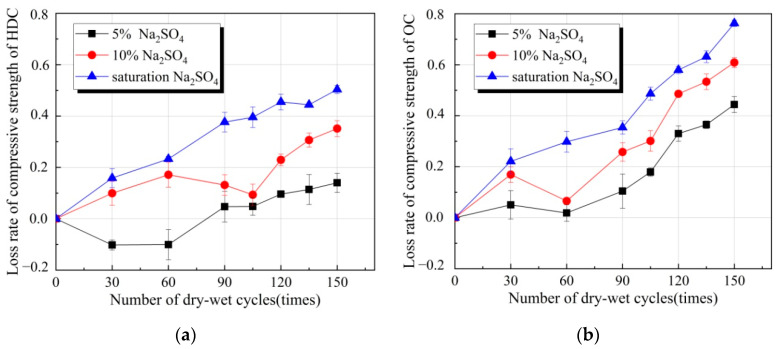
Loss rate of compressive strength of HDC and OC under different times of dry–wet cycles in different concentration sulfate solutions. (**a**) HDC; (**b**) OC.

The relationship between the loss rate of the compressive strengths of HDC and OC and the number of dry–wet cycles under different concentrations of sulfate solution are shown in Figure 2. As seen in Figure 2, as the number of dry–wet cycles increased, the loss rate of the compressive strengths of HDC and OC gradually increased. With the increase in the concentration of sulfate solution, the loss rate of the compressive strengths of HDC and OC gradually increased at the same number of dry–wet cycles. The loss rate of the compressive strength was a positive value, indicating that the compressive strengths of HDC and OC decreased, and the HDC and OC were damaged by sulfate attack. Otherwise, the compressive strength increased.

Figure 2a shows that under the 5% sulfate solution, the loss rate of the compressive strength of HDC before 90 dry–wet cycles was negative, indicating that the compressive strength of HDC under sulfate attack is greater than the compressive strength of HDC in water under the same number of wet and dry cycles, but the loss rate of the compressive strength of HDC after 90 dry–wet cycles was positive and its value is 4.7% at 90 dry–wet cycles and 14.0% at 150 dry–wet cycles. With the increase in the number of dry–wet cycles, the loss rate of the compressive strength gradually increased. However, the compressive strength gradually decreased. With the 10% and saturated sulfate solution, the loss rate of the compressive strength of HDC was positive. For example, the loss rate of the compressive strength was 50.3% with 150 dry–wet cycles under the saturated sulfate solution, indicating that it was damaged by sulfate attack. A higher concentration of the sulfate solution entailed a greater loss rate of the compressive strength of HDC, indicating that the degree of damage was more serious. With the increase in the number of dry–wet cycles, the loss rate of the compressive strengths of HDC and OC generally showed an increasing trend. Under the same number of dry–wet cycles, the loss rate of the compressive strengths of HDC and OC gradually increased as the concentration of sulfate solution increased, and the OC was greater than the HDC. Under the same sulfate concentration and the same number of dry–wet cycles, the loss rate of the compressive strength of OC was greater than that of HDC. For example, when the dry–wet cycles were 150 times in the saturation, the loss rate of the compressive strength of OC was 76.3%, while that of HDC was 50.3%.

## 4. Damage Mechanism of HDC

### 4.1. Experimental Phenomena

**Figure 3:**
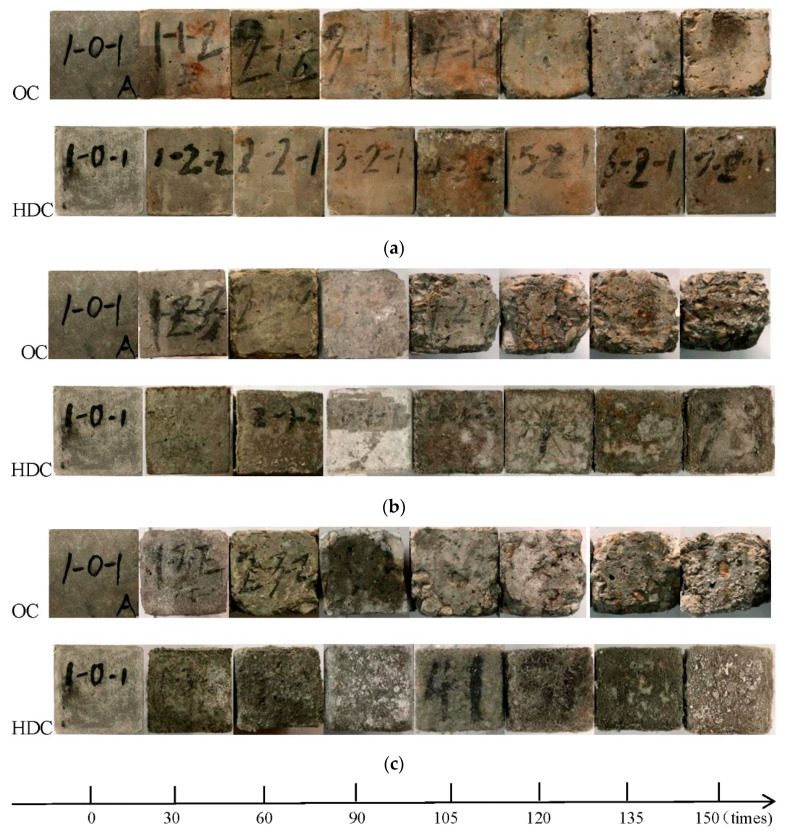
Morphology of HDC and OC samples with different times of dry–wet cycles in sulfate solution:(**a**) 5% sulfate solution; (**b**) 10% sulfate solution; (**c**) saturated sulfate solution.

The morphologies of the OC and HDC samples with different numbersof dry–wet cycles under the action of sulfate solution are shown in Figure 3. As seen in Figure 3, under the 5% sulfate solution, the HDC sample could maintain its complete shape after 150 dry–wet cycles. The matrix had no shedding, no crystals were present on the surface, and no fibers were pulled out, but cracks on the surface were evident. The corrosion resistance coefficient (which refers to the ability of the sample to resist sulfate attack) is defined as *K*_f_ = *f_c_*_,n_/*f_c_*_,0_, where *f_c_*_,n_ and *f_c_*_,0_ are the compressive strength of *n* dry–wet cycles in different concentrations of sulfate solution and the compressive strength of non-dry–wet cycle samples, respectively. The corrosion resistance coefficient of the sample was 1.09, which could still work normally, indicating that HDC had strong resistance to sulfate attack at this time. However, after 150 dry–wet cycles, the OC sample was no longer a complete cube, and its coarse aggregate was exposed. The compressive strength of OC was significantly reduced, indicating that it was unable to resist sulfate attack at this time.

As shown in Figure 3, under the 10% sulfate solution, after 60 dry–wet cycles, pitting corrosion occurred on the surface of the HDC sample. After 150 dry–wet cycles, the surface fiber was exposed, and the fiber adhered to the cement-based fragment; however, the sample retainedits shape. Given the presence of the PVA fiber, the crack propagation speed of HDC was slower than that of the OC. HDC had no coarse aggregate, and its particles were finer. The specific surface area was larger, the structure was denser, and the original pores were smaller, resulting in the number of formation ettringite being relatively small compared with that of OC. The volume expansion rate and the internal stress were small, and the number of cracks was relatively lower. After 60 dry–wet cycles, the OC sample showed angular detachment. After 150 cycles, the OC surface matrix completely fell off. The shape was extremely irregular, the aggregate was exposed, and the crystallization was serious. The OC sample was seriously damaged.

As shown in Figure 3, under the saturated sulfate solution, the fiber of the HDC sample was exposed after 60 dry–wet cycles; the volume of HDC sample expanded, and the shape of HDC remained complete. After 150 dry–wet cycles, the HDC compressive strength was severely lost. HDC could not be used normally; a corner drop was present, the surface was crystallized, and the volume expanded. However, after 60 dry–wet cycles, the OC evidently had angular detachment, and the internal coarse aggregate was exposed. At 150 times dry–wet cycles, the corrosion resistance coefficient was 0.63, indicating that the compressive strength of OC was seriously lost, and the bearing capacity of OC was lost. Therefore, HDC has the ability to resist sulfate attack and dry–wet cycles and is superior to OC.

### 4.2. Physical and Chemical Damage Mechanism

The numerous ions of the cement-based composite material in the sample and SO_4_^2^^−^ in the sulfate solution underwent a series of physical and chemical reactions. Thus, the interior of the concrete and the cement matrix were eroded, and the structural pores changed, thus resulting in changes in mechanical properties.

**Figure 4:**
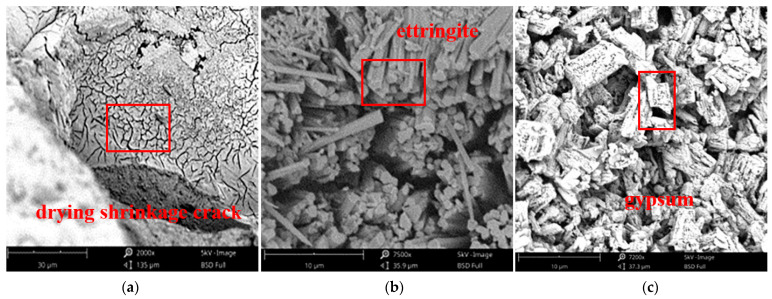

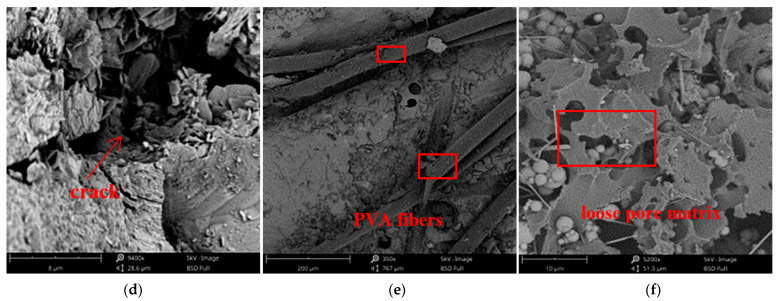
Microstructureof HDC. (**a**) Drying shrinkage crack (30μm); (**b**) ettringite (10μm); (**c**) gypsum (10μm); (**d**) crack (8μm); (**e**) PVA (200μm); (**f**) loose pore matrix (10μm).

The SEM scanning microstructure of the sample is shown in Figure 4. Under the action of the dry–wet cycles, HDC lost water and shrank when drying, resulting in small cracks (Figure 4). With the increase in the number of dry–wet cycles, the cracks became increasingly large. In the sulfate solution, the cement hydration product reacted with SO_4_^2^^−^ to produce ettringite and gypsum. Their biggest characteristic was expansibility. The volume expansion was approximately 120%, and the volume expansion generated internal stress. When the internal stress was greater than the strength of the matrix, cracks appeared inside the sample, thus forming pores, which was conducive to the transmission and penetration of SO_4_^2^^−^. Before 60 dry–wet cycles, the quality of HDC continued to increase, indicating that gypsum, ettringite formation, or sulfate crystals precipitated and filled the pores, resulting in an increase in mass; thus, its compressive strength also increased (Figure 1). With the increase in the concentration of the sulfate solution and the number of dry–wet cycles, the chemical reaction was intensified, and the generated gypsum or ettringite or precipitated sulfate crystals increased, resulting in larger and more cracks in the HDC. These cracks eventually flaked off, resulting in quality and strength loss. The degree of HDC damage was lower than that of OC, as HDC was doped with PVA fiber (Figure 4). PVA fiber had the functions of crack resistance, toughening, and reinforcement, which prevented the rapid development of cracks and delayed the rate of sulfate attack.

The main hydration products of cement were C-S-H, C-H, C-A-H, AFt, and AFm. The hydration product under the sulfate solution reacted as follows [37,38,39]:

(1) $$2Na^{+}+SO_{4}{}^{2-}+10H_{2}O\Leftrightarrow Na_{2}SO_{4}\cdot 10H_{2}O$$

(2) $$\left\{ \begin{array}{c} 3CaO\cdot Al_{2}O_{3}+3\left(CaSO_{4}\cdot 2H_{2}O\right)+26H_{2}O\to 3CaO\cdot Al_{2}O_{3}\cdot 3CaSO_{4}\cdot 32H_{2}O\\ 3CaO\cdot Al_{2}O_{3}+CaSO4\cdot 12H_{2}O+2\left(CaSO_{4}\cdot 2H_{2}O\right)+16H_{2}O\to 3CaO\cdot Al_{2}O_{3}\cdot 3CaSO_{4}\cdot 32\;H_{2}O\\ 4CaO\cdot Al_{2}O_{3}\cdot 13H_{2}O+3\left(CaSO_{4}\cdot 2H_{2}O\right)+14H_{2}O\to 3CaO\cdot Al_{2}O_{3}\cdot 3CaSO_{4}\cdot 32H_{2}O+CaO\cdot H_{2}O\\ Ca\;{\left(OH\right)}_{2}+Na_{2}SO_{4}+2H_{2}O\to CaSO_{4}\cdot 2H_{2}O+2NaOH \end{array}\right.$$

The chemical reaction formula between cement hydrate and sulfate solution is presented in Equations (1) and (2). Given the defects of concrete, such as pores and cracks, SO_4_^2−^ was transmitted into HDC through pores and cracks and reacted with the hydration products of cement, forming ettringite and gypsum. Gypsum is the product of a high concentration of SO_4_^2−^. It filled in the pores and cracks of the sample and had a certain repair effect on the structural defects by improving the compactness and compressive strength. According to crystallization theory, when the sulfate solution reaches the supersaturation state, the sulfate crystallizes and precipitates [40]. Therefore, during the dry–wet cycles, with the evaporation of water, the sulfate salt crystallization (Na_2_SO_4_·10H_2_O) precipitated, which was distributed inside or on the surface of the sample. As the number of dry–wet cycles increased, cracks and pores increased, the matrix became loose, and the compressive strength was lost (Figure 1 and Figure 4).

### 4.3. Destruction Mechanism of HDC under Uniaxial Compression

**Figure 5:**
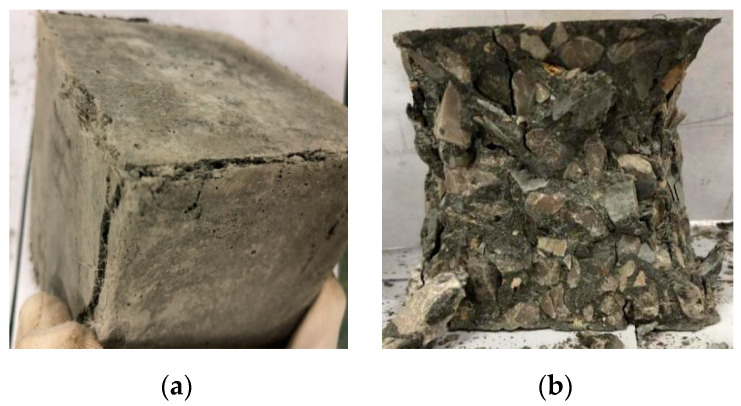
Failure modes of HDC and OC under compression. (**a**) HDC; (**b**)OC.

The uniaxial compression failure modes of HDC and OC are shown in Figure 5. In the compressive strength test, the PVA fibers were evenly distributed in the HDC sample, similar to the short reinforced arrangement. Given the crack resistance and toughening effect of the PVA fiber, the development of HDC cracks was prevented, and the ductility of HDC increased under uniaxial compression. The microcracks first appeared in the compression direction. With the increase in the load, the cracks continued to expand and increase, and the cement matrix produced penetrating cracks. When the load continued to increase, the fiber assumed part of the load until the fiber was broken or pulled out, showing a failure mode with a “drum shape”. The HDC sample was complete and cracked, but not scattered, which was similar to ductile damage, which is also similar to that of the eccentric compression column researched by Deng [41]. The middle of the OC sample swelled and fell off, forming failure modes of two inverted cones with a “slim waist” shape, which was considered a brittle failure [11,34].

### 4.4. Damage Variables of HDC

The damage variable is defined ascompressive strength. Based on phenomenological method theory, the damage variable under different dry–wet cycles and different concentrations of sulfate solution was calculated using Equation (3) [41,42,43,44]:

(3) $$D\left(n\right)=1-\frac{f_{c,}{}_{n}}{f_{c,}{}_{0}}$$

where *D*(*n*) is the damage variable under different times of dry–wet cycles. *f_c_*_,n_ is the compressive strength of the sample after *n* dry–wet cycles (MPa). *f_c_*_,0_ is the compressive strength of the sample at 0 dry–wet cycles for 28 days of standard curing (OC) or 90 days of standard curing (HDC), *f_c_*_,0_ = 45.8 MPa (HDC) or 52 MPa (OC).

**Figure 6:**
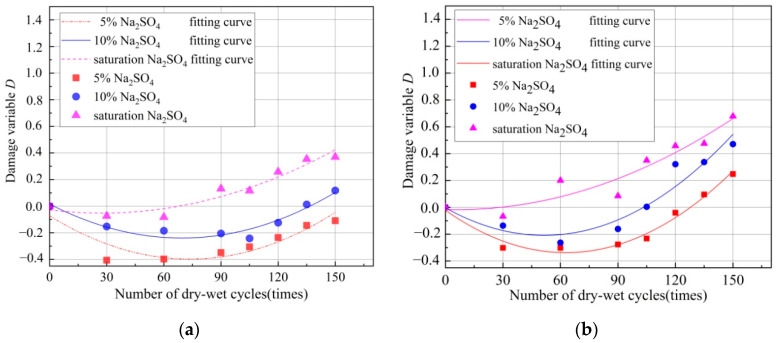
Relationship between damage variables of OC and HDC and number of dry–wet cycles in different concentrations of sulfate solution. (**a**) *D* of HDC; (**b**) *D* of OC.

The damage variables of HDC and OC under different concentrations of sulfate solution and different numbers of dry–wet cycles are shown in Figure 6. The damage variable was negative, indicating that the compressive strength increased. The damage variable was positive, the material was damaged, and the compressive strength decreased. As seen in Figure 6, with the increase in the number of dry–wet cycles, the damage variable of the HDC sample first showed a negative value and gradually increased in absolute value; then, it presented a positive value and gradually increased. The damage variables of the HDC samples under the 5% sulfate solution were all negative values, indicating that the compressive strength increased. After 30 dry–wet cycles, the damage variables tended to increase. However, the damage variables were still negative, indicating that the compressive strength was gradually reduced but still greater than the initial value. Therefore, the damage of the HDC sample under the 5% sulfate solution was not evident. At the 10% and saturated sulfate solution, the damage variable was first negative and then positive, indicating that the compressive strength first increased and then decreased. As the number of dry–wet cycles increased, the damage increased. Thus, the damage to the HDC sample under the 10% and saturated sulfate solution was obvious. As the concentration of the sulfate solution increased, the damage of the HDC sample intensified. The overall change trend of OC was similar to that of HDC, but its change was more evident than that of HDC.

The damage variables of HDC and OC were respectively fitted with polynomial curves, and the functional relationship curves of the damage variables at different concentrations of the sulfate solution with the number of dry–wet cycles are shown in Figure 6. The two can be uniformly expressed as:

*D*(*n*) = *an*^2^ + *bn*+ *c* (4)

where *D*(*n*) is the damage variable under different times of dry–wet cycles. *a*, *b*, and *c* are the coefficients related to the chemical solution and its concentration under the action of the dry–wet cycles. *n* is the number of dry–wet cycles.

According to the results of the experiment, the fitting parameters of HDC and OC are shown in Table 6. The fitting curve shows that the damage variable changed the quadratic polynomial positively with the number of dry–wet cycles. According to Formula (4), the damage variables of the chemical solutions of different concentrations under different times of dry–wet cycles could be calculated to characterize the degree of damage. As seen in Figure 6 and Table 6, a certain error existed between the experimental value and the individual value of the fitted value. The reasons were as follows. First, the HDC had difficulty achieving uniform fiber distribution during stirring. With the increase in the number of dry–wet cycles and the increase in sulfate concentration, the HDC damage increased, resulting in an increase in the difference between the fitted value and the test value. Second, fewer test data were used, which affected the fitting parameters. Third, the internal reasons of HDC and the damage and errors caused by different samples of the same batch in the test resulted in a certain degree of dispersion in the test data. However, the comparison found that the experimental value and the fitting value were consistent. Therefore, the damage toHDC was not only related to the number of dry–wet cycles but also closely related to the concentration of the sulfate solution. Both factors interacted with each other and affected the HDC together.

## 5. Conclusions

In this study, through the experimental study of the mechanical properties of HDC under the combined action of sulfate attack and dry–wet cycles, the following conclusions are drawn:

(1) HDC has toughening and crack resistance, reduces the occurrence of cracks, and increases compactness. According tothe compressive strength test, the failure mode of HDC is drum-shaped with cracks was but not loose—similar to ductile failure—which effectively overcomes the brittle failure characteristics of OC.
(2) With the increase in the number of dry–wet cycles, the compressive strength of HDC generally increases first and then decreases. Before 30 dry–wet cycles, the compressive strength of HDC gradually increased. After 105 dry–wet cycles, the compressive strength of HDC decreasedin a straight line, and the loss rate of the compressive strength of HDC generally increased. Under the same concentration of sulfate solution and the number of dry–wet cycles, the loss rate of the compressive strength of HDC is less than OC. As indicated above, the ability of HDC to resist dry–wet cycles is better than that of OC.
(3) As the concentration of the sulfate solution increases, the max compressive strength of HDC gradually decreases; that is, the damage is intensified. The 5% sulfate solution resulted inno evident damage to HDC, and 10% and saturated solutions resulted inserious damage to the HDC. HDC damage is the result of the combined action of sulfate attack and dry–wet cycles. Comparison shows that HDC has the ability to resist sulfate attack and dry–wet cycles and is superior to OC.

## Figures and Tables

**Table 1:** Performance indicatorsof PVA.

Length/mm	Diameter/μm	Tensile Strength/MPa	Elastic Modulus/GPa	Elongation/%
12.0	39.0	1620.0	42.8	7.0

**Table 2:** Mix proportion of HDC and OC.

Specimen Type	Mix Proportion/L						
	Cement/g	Fly Ash/g	Sand/g	Gravel/g	Water/mL	Water Reducer/mL	PVA/g
HDC	598.2	598.2	430.7	0.0	346.9	2.5	26.0
OC	514.0	0.0	541.0	1205.0	165.0	7.7	0.0

**Table 3:** Chemical composition of fly ash and cement.

Composition	Fly Ash	Cement
SiO_2_ (%)	42.60	21.38
Al_2_O_3_ (%)	26.41	5.91
CaO (%)	5.57	60.16
Fe_2_O_3_ (%)	4.22	2.71
SO_3_ (%)	0.28	1.96
MgO (%)	0.83	1.25
TiO_2_ (%)	1.17	0.36
Alkalis (%)	1.80	0.30
P_2_O_5_ (%)	0.20	0.44
f-CaO (%)	0.47	0.91

**Table 4:** The compressive strength of HDC.

Times	5% Na_2_SO_4_				10% Na_2_SO_4_				Saturation Na_2_SO_4_				Water			
	Compressive Strength/MPa			Mean	Compressive Strength/MPa			Mean	Compressive Strength/MPa			Mean	Compressive Strength/MPa			Mean
0	46.1	45.3	45.9	45.8	46.1	45.3	45.9	45.8	46.1	45.3	45.9	45.8	46.1	45.3	45.9	45.8
30	66.2	65.9	61.2	64.4	52.2	58.4	47.7	52.8	51.8	47.8	47.9	49.2	59.7	61.2	54.6	58.5
60	62.1	60.6	69.4	64.0	52.8	48.4	43.6	48.3	45.2	43	45.6	44.6	55.2	59.5	59.8	58.2
90	57.8	59.4	64.2	60.5	54.8	50.6	60.3	55.2	44.8	34.4	40.1	39.8	57.2	66.6	67.1	63.6
105	54.7	61	63.8	59.8	52.4	59.2	59.2	56.9	33.6	39.9	40.5	38.0	66.7	62	59.7	62.8
120	61.3	57.7	50.7	56.6	41.9	49.8	53.2	48.3	32.7	33.5	35.8	34.0	67.8	56.3	63.6	62.6
135	48.7	53.9	54.7	52.4	43.9	42.4	37.1	41.1	37.2	33.4	28.7	33.1	66.1	60.8	51.6	59.5
150	49.7	51.9	48.6	50.1	42.1	33.2	38.5	37.9	27.6	30.7	28.5	28.9	62.0	59.2	53.8	58.3

**Table 5:** The compressive strength of OC.

Times	5% Na_2_SO_4_				10% Na_2_SO_4_				Saturation Na_2_SO_4_				Water			
	Compressive Strength/MPa			Mean	Compressive Strength/MPa			Mean	Compressive Strength/MPa			Mean	Compressive Strength/MPa			Mean
0	48.0	52.2	55.6	51.9	48.0	52.2	55.6	51.9	48.0	52.2	55.6	51.9	48.0	52.2	55.6	51.9
30	74.5	67.3	61.3	67.7	53.4	59.1	52.2	54.9	50.8	54.2	61.6	55.5	67.9	73.7	72.0	71.2
60	66.6	67.9	68.7	67.7	71.1	68.2	65.7	68.3	43.3	49.8	52.6	48.6	68.5	66.1	72.7	69.1
90	74.1	66.4	58	66.2	50.1	60.4	53.8	54.8	43.5	49.7	49.7	47.6	70.1	77.1	73.9	73.7
105	61.5	66.3	64.4	64.1	59.4	52.5	51.8	54.6	38.5	38.8	42.9	40.1	80.7	80.1	73.5	78.1
120	49.7	55.1	57.4	54.1	45	41.1	38.5	41.5	38.8	33.3	30.1	34.1	89.6	78.7	74.3	80.9
135	42.6	48.1	50.4	47.0	37	33.2	33.2	34.5	30.2	26.5	25.1	27.3	77.3	77.0	67.8	74.0
150	35.2	40.2	42	39.1	30	25.3	27.3	27.5	18.3	15.5	16.3	16.7	72.7	70.5	67.7	70.3

**Table 6:** Fitting parameters of HDC and OC.

Specimen Type	Fitting Parameters				
	Na_2_SO_4_	*a*	*b*	*c*	*R* ^2^
HDC	5%	6.016 × 10^−5^	−0.009	−0.072	0.815
	10%	5.293 × 10^−5^	−0.007	0.016	0.919
	saturation	3.172 × 10^−5^	−0.002	−0.028	0.932
OC	5%	7.995 × 10^−5^	−0.010	−0.022	0.975
	10%	7.746 × 10^−5^	−0.008	−0.004	0.917
	saturation	3.271 × 10^−5^	−0.0004	−0.016	0.908

## Data Availability

The data presented in this study are available in the article.
